# CXCR4 expression in feline mammary carcinoma cells: evidence of a proliferative role for the SDF-1/CXCR4 axis

**DOI:** 10.1186/1746-6148-8-27

**Published:** 2012-03-14

**Authors:** Angelo Ferrari, Claudio Petterino, Alessandra Ratto, Chiara Campanella, Roberto Wurth, Stefano Thellung, Guendalina Vito, Federica Barbieri, Tullio Florio

**Affiliations:** 1 Istituto Zooprofilattico Sperimentale del Piemonte, Liguria e Valle D'Aosta, National Reference Center of Veterinary and Comparative Oncology (CEROVEC), Piazza Borgo Pila, 16129 Genova, Italy; 2 Section of Pharmacology, Department of Internal Medicine, and Centre of Excellence for Biomedical Research, University of Genova, Viale Benedetto XV 2, 16132 Genova, Italy

## Abstract

**Background:**

Mammary tumours frequently develop in female domestic cats being highly malignant in a large percentage of cases. Chemokines regulate many physiological and pathological processes including organogenesis, chemotaxis of inflammatory cells, as well as tumour progression and metastasization. In particular, the chemokine/receptor pair SDF-1/CXCR4 has been involved in the regulation of metastatic potential of neoplastic cells, including breast cancer. The aim of this study was the immunohistochemical defininition of the expression profile of CXCR4 in primary and metastatic feline mammary carcinomas and the evaluation of the role of SDF-1 in feline mammary tumour cell proliferation.

**Results:**

A total of 45 mammary surgical samples, including 33 primary tumours (31 carcinomas and 2 adenomas), 6 metastases, and 4 normal mammary tissues were anlyzed. Tumor samples were collected from a total number of 26 animals, as in some cases concurrent occurrence of neoplasm in more than one mammary gland was observed. Tissues were processed for standard histological examination, and all lesions were classified according to the World Health Organization criteria. CXCR4 expression in neoplastic cells was evaluated by immunohistochemistry. The level of CXCR4 immunoreactivity was semi-quantitatively estimated as CXCR4 score evaluating both the number of positive cells and the intensity of staining. Six primary, fibroblast-free primary cultures were obtained from fresh feline mammary carcinomas and characterized by immunofluorescence for CXCR4 and malignant mammary cell marker expression. SDF-1-dependent *in vitro *proliferative effects were also assayed. CXCR4 expression was observed in 29 out of 31 malignant tissues with a higher CXCR4 score observed in 4 out of 6 metastatic lesions than in the respective primary tumours. In 2 benign lesions analyzed, only the single basaloid adenoma showed a mild positive immunostaining against CXCR4. Normal tissue did not show CXCR4 immunoreactivity. CXCR4 score was statistically significantly associated with the histological features of the samples, showing an increase accordingly with the degree of neoplastic transformation (from normal tissue to metastatic lesions). Finally, in the primary cultures obtained from 6 primary feline mammary carcinomas CXCR4 expression was detected in all cells and its activation by SDF-1 in vitro treatment caused a significant increase in the proliferation rate in 5 out of 6 tumours.

**Conclusions:**

These results indicate that malignant feline mammary tumours commonly express CXCR4, with a higher level in malignant tumours, and, in most of the cases analysed, metastatic cells display stronger immunoreactivity for CXCR4 than the corresponding primary tumours. Moreover, CXCR4 activation in primary cultures of feline mammary carcinomas causes increase in the proliferative rate. Thus, SDF-1/CXCR4 system seems to play a tumorigenic in feline mammary gland malignancy and in vitro cultures from these tumour samples may represent an experimental model to investigate the biological and pharmacological role of this chemokinergic axis.

## Background

Chemokines are small messengers with chemoattractant function (chemotactic cytokines). They belong to a large superfamily of peptides produced and secreted by different cell types and classified in four groups (CC, CXC, C, and CX3C) accordingly to structural determinants [1]. The complex chemokine system is involved in a wide range of cell functions ranging from organogenesis to malignancy. Chemokine activity is mediated by the activation of a family of specific G protein coupled receptors. Chemokine receptor activation is mediated by coupling to intracellular heterotrimeric G-proteins associated with the inner surface of the plasma membrane [2]. Upon ligand binding, chemokine receptors promote G protein activation leading to the inhibition of cAMP synthesis and the activation of phospholipase C that cleaves phosphatidylinositol (4,5)-bisphosphate (PIP_2_) into the second messengers inositol triphosphate (IP_3_) and diacylglycerol (DAG). DAG activates protein kinase-C (PKC), while IP_3 _induces the release of calcium ions from intracellular stores. Several studies supported the role of chemokinergic axis in physiological activities including organogenesis [3], haematopoiesis [4], angiogenesis [5], homing of lymphocytes [6], immune response and inflammation [7].

Among CXC chemokine receptors, in the past years, CXCR4 attracted great attention for its pleiotropic activity outside the immune system [8,9]. CXCR4 activity is dependent on its interaction with its unique ligand: stromal cell-derived factor-1 (SDF-1, also named CXCL12) [10]. SDF-1 was recently reported to bind also a second receptor CXCR7, which regulates very different cellular activities [11] including tumor angiogenesis [12-14]. Beside regulation of Ca^++ ^homeostasis and PKC activation, CXCR4 also modulate ERK1/2 MAP kinase and Akt activities through a paracrine/autocrine mechanism [15,16]. The activation of all these signalling cascades generates specific biological responses, such as chemotaxis, degranulation, release of superoxide anions, and cell proliferation. Over-expression of CXCR4 is considered a key regulatory step in several human malignancies included breast cancer, resulting in a poor prognosis [17,18]. In detail, CXCR4 activity was involved in the oestrogen resistance of breast cancer [19]. Moreover, high CXCR4 expression has been related to the metastatic potential of breast cancer cells, since. *in vitro *experiments showed that CXCR4 activation by SDF-1 regulates motility and metastatic potential of neoplastic epithelial cell lines [20,21], while CXCR4 inhibitors are effective in reducing chemotactic and metastatic potential of this receptor [20].

In cats, most studies are focused on the characterization of the CXCR4 role associated with FIV infection [22,23], because of the role of this receptor as mediator of virus cell entry, as also observed in humans [24]. Conversely, few studies have been targeted on the role of CXCR4 in feline mammary gland neoplasia [25,26], although CXCR4 may have a role in development of mammary carcinoma in cats as in women. Feline mammary tumors show age, incidence, histopathology and pattern of metastasis similar to human breast cancer [27]. In addition, the lack of estrogens dependence in most of these tumours, suggests that this cancer may represent a suitable animal model for estrogen receptor (ER) negative breast cancer [28]. Recently, feline mammary carcinoma subtypes have been described to share features with human inflammatory mammary carcinoma [29]. At the molecular level HER2 overexpressing malignancies in cats may be considered homologue of the HER2 positive- poor prognosis-human counterpart [30].

Thus, research in this field may be relevant to better understand the biology of feline mammary tumour, also as a comparative model for human breast cancer, and to evaluate the CXCR4 role in cancer mechanisms. Moreover, immunohistochemistry screening of feline mammary tumours may have interesting applications in clinical practice as prognostic factor and to evaluate the possible response to CXCR4 inhibitors.

The aim of the present study was to investigate, by immunohistochemistry, the levels of CXCR4 expression in feline mammary tumours and metastases, and the proliferative activity induced by SDF-1 on feline carcinoma primary cultures.

## Results

### Feline mammary tumours and histological diagnoses

The clinical and pathological data of the feline mammary carcinomas analysed are summarised in Table 1. The 26 cats under study included 25 domestic short-hairs and 1 persian; 9 were intact females, while 17 were spayed. The age ranged from 5 to 19 years (median age, 12 years). In one case the age was not available. Primary malignant tumours were observed in 24/26 cats (92%), 7/26 animals beard two neoplastic mammary glands thus we collected a total of 31 primary tumour samples. Only 2/26 benign tumours were observed (8%). The primary tumor samples under evaluation included 14 simple tubulopapillary primary carcinomas (STPCs), of which four of them gave metastasis (Table 1). The other samples were diagnosed as solid carcinomas (SCs, 7 cases) of which one gave lymph node metastasis, 6 cribriform carcinomas (CCs), 3 simple tubular carcinomas (STCs) and 1 metastatic adenosquamous carcinoma (ASC). (Table 1). Twenty-seven out of 31 primary malignant lesions were classified as high grade carcinomas (grade III, 87%) and 4 as intermediate grade (grade II, 13%). No grade I tumours were observed in this study. Only 2 benign tumours (1 basaloid adenoma, and 1 complex adenoma) were diagnosed in this group of animals. Four normal tissue samples, derived from non-neoplastic mammary glands resected during tumor surgery, were included in the study as CXCR4 expression reference.

**Table 1:** Clinico-pathological features of feline mammary tissues

Cat **n**.	Sample n.*	Sex	Age (yrs)	Breed	Histology	Grade	CXCR4 score
1	1a	F	13	DSH	STPC	II	3+

	1b				STPC	II	1+

**2**	**2**	**FS**	**14**	**DSH**	**STPC**	**III**	**2+**

	**2M**				**metastasis (adipose tissue)**		**2+**

3	3a	FS	7	DSH	STPC	III	1+

	3b				STPC	III	3+

**4**	**4a**	**FS**	**15**	**DSH**	**STPC**	**III**	**2+**

	**4aM**				**metastasis (lymph node)**		**3+**

	4b				STPC	III	2+

5	5a	F	10	DSH	STPC	III	1+

	**5b**				**STPC**	**III**	**2+**

	**5bM**				**metastasis (lymph node)**		**3+**

6	6a	FS	12	DSH	STPC	III	2+

	6b				STPC	III	2+

7	7a	F	15	DSH	STPC	III	2+

	7b				STPC	III	2+

**8**	**8**	**FS**	**12**	**DSH**	**STPC**	**II**	**3+**

	**8M**				**metastasis (lymph node)**		**1+**

9	9a	F	8	DSH	SC	III	1+

	9b				SC	III	0

10	10	F	14	DSH	SC	III	2+

11	11	FS	9	DSH	SC	III	3+

**12**	**12**	**FS**	**14**	**DSH**	SC	**III**	**2+**

	**12M**				**metastasis (lymph node)**		**3+**

13	13	F	10	DSH	SC	III	3+

14	14	F	5	DSH	SC	III	1+

15	15	F	19	PC	CC	III	2+

16	16	FS	13	DSH	CC	III	3+

17	17	FS	9	DSH	CC	III	1+

18	18	FS	14	DSH	CC	III	1+

19	19	FS	9	DSH	STC	II	2+

20	20	FS	n.a	DSH	STC	III	2+

21	21	F	12	DSH	STC	III	0

22	22	FS	11	DSH	CC	III	3+

**23**	**23**	**FS**	**19**	**DSH**	**ASC**	**III**	**2+**

	**23M**				**metastasis (lung)**		**3+**

24	24	FS	12	DSH	CC	III	2+

25	25	FS	4	DSH	BA	-	1+

26	26	FS	8	DSH	CA	-	0

27	27	FS	12	DSH	NORMAL (M4 D)	-	0

28	28	FS	19	DSH	NORMAL (M1 S)	-	0

29	29	FS	11	DSH	NORMAL (M4 D)	-	0

30	30	F	8	DSH	NORMAL (M2 S)	-	0

F, female, FS, female spayed; DSH, domestic short-hair; PC, persian; STPC, simple tubulopapillary carcinoma; SC, solid carcinoma; STC, simple tubular carcinoma; CC, cribriform carcinoma; BA, basaloid adenoma; CA, complex adenoma; ASC, adenosquamous carcinoma; n.a., not available. For normal tissues the mammary gland topography is indicated. *a and b indicate samples from two glands of the same cat, M labels metastatic tissue. Primary tumours and corresponding metastases of the same cat are reported in bold.

### CXCR4 expression in feline mammary carcinomas

**Figure 1:**
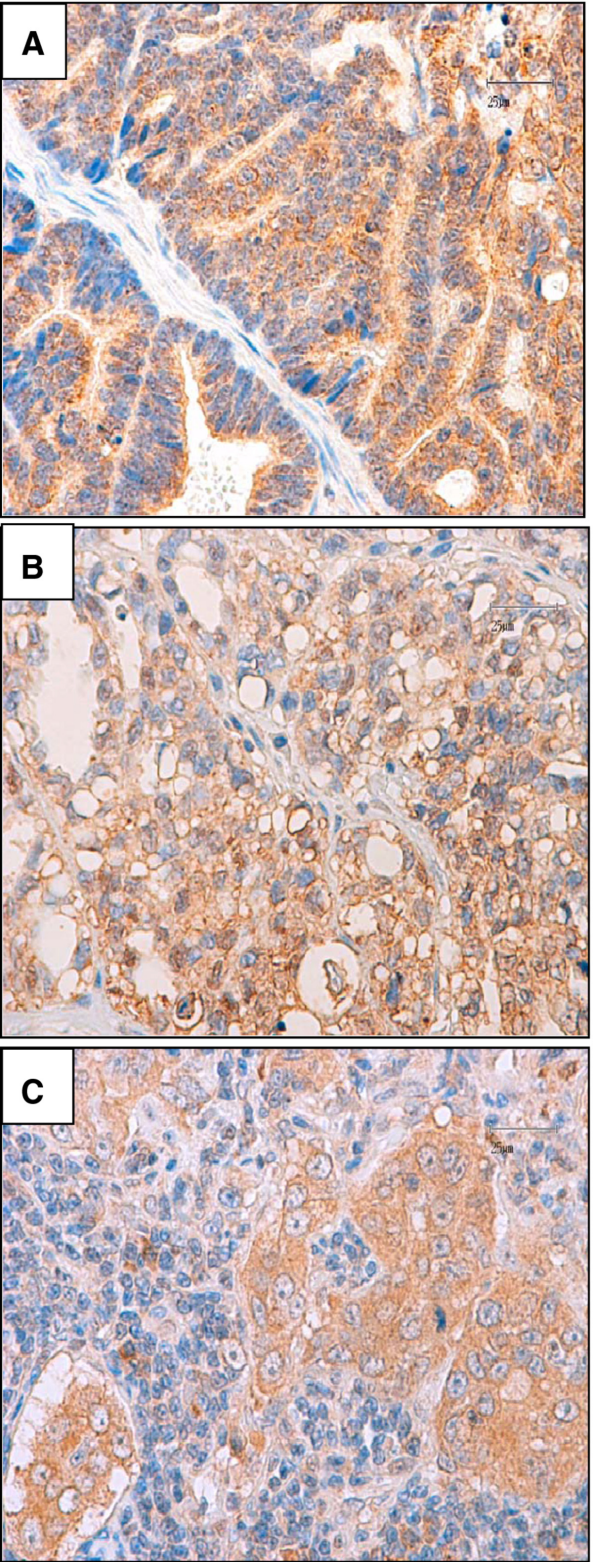
**Immunohistochemical analysis of CXCR4 expression in feline mammary carcinomas**. **Panel A**. Representative primary feline carcinoma showing a high number of neoplastic cells with moderate to intense cytoplasmic positivity for CXCR4. (Bar 25 μm). **Panel B**. Primary tubulopapillary carcinoma: a poorly differentiated area with low number of positive neoplastic cells showing a weak immunoreactivity for CXCR4. (Bar 25 μm). **Panel C**. Lymph node metastasis of the feline mammary carcinoma showed in Panel B. Metastatic cells show a marked immunoreaction for CXCR4 if compared with the corresponding primary lesion. (Bar 25 μm). Original magnification 40×.

The expression of CXCR4, analyzed by immunohistochemistry, was observed in 29 out of 31 (93%) of primary carcinoma samples (from a total of 24 queens) although the staining pattern and intensity differed between tumors as evaluated by CXCR4 scores (1+, 2+ and 3+) reported in Table 1. In 7 out of 24 carcinoma-bearing animals we analyzed tumors taken from two different mammary glands of the same cat (named as a and b in Table 1); in this subgroup 4/7 tumors showed different score of CXCR4 expression among carcinomas derived from gland a and b from the same cat, possibly reflecting heterogeneity across tumour lesions. The expression of CXCR4 was mostly confined to the membrane and cytoplasm of neoplastic cells (Figure 1) and differed in intensity and staining pattern (Figure 1, panels A and B) and among primary (Figure 1, panel B) and, in most cases, the corresponding metastatic lesions (Figure 1, panel C). Negative controls and CXCR4 antibody specificity were performed as reported in supplementary Figure S1.

**Figure 2:**
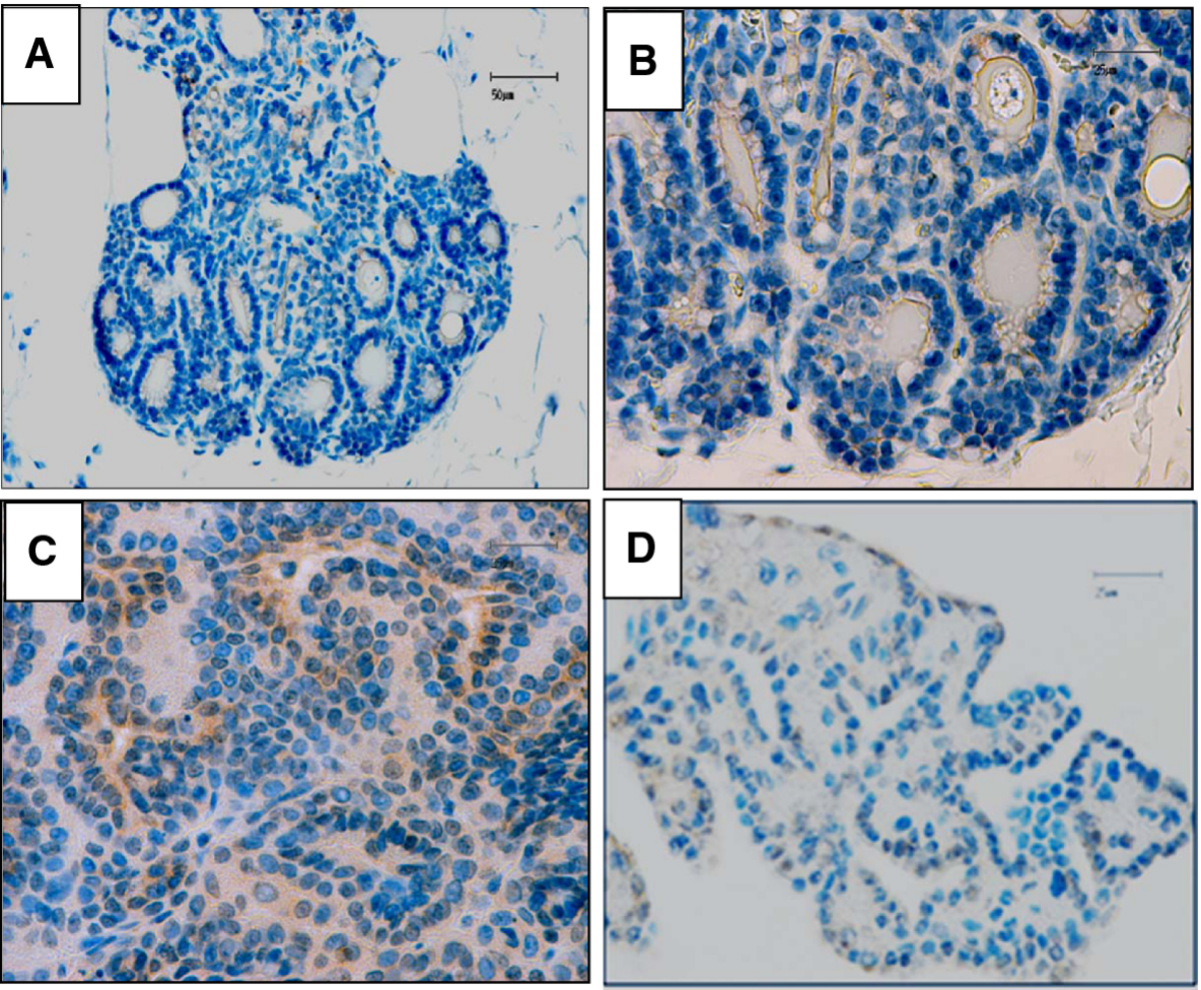
**Immunohistochemical analysis of CXCR4 expression in normal feline mammary gland and benign lesions**. **Panel A**. Representative photomicrograph of a normal lobule of feline mammary gland tissue showing CXCR4 negativity (Bar 50 μm, original magnification 20×). **Panel B**. Detail of the normal lobule of feline mammary gland tissue showing several CXCR4-negative acini, (Bar 25 μm, original magnification 40×). **Panel C**. Feline benign mammary lesion (basaloid adenoma, cat n. 25): epithelial cells show negative or weak immunoreaction for CXCR4 (Bar 25 μm, original magnification 40×). **Panel D**. Feline benign mammary lesion (complex adenoma, cat n. 26): negative immunoreaction for CXCR4. (Bar 25 μm, original magnification 40×).

To evaluate the specificity of CXCR4 expression in mammary carcinomas and metastasis, we analyzed the level of CXCR4 immunopositivity in normal mammary tissues and benign lesions (1 basaloid adenoma and 1 complex adenoma). As depicted in Figure 2 (panels A and B), normal mammary gland tissue did not express detectable level of CXCR4, while epithelial cells of basaloid adenoma appeared weakly positive for CXCR4 (CXCR4 score 1+, Figure 2 panel C) in scattered areas throughout the section, and the complex adenoma was essentially negative (CXCR4 score 0, Figure 2 panel D).

**Figure 3:**
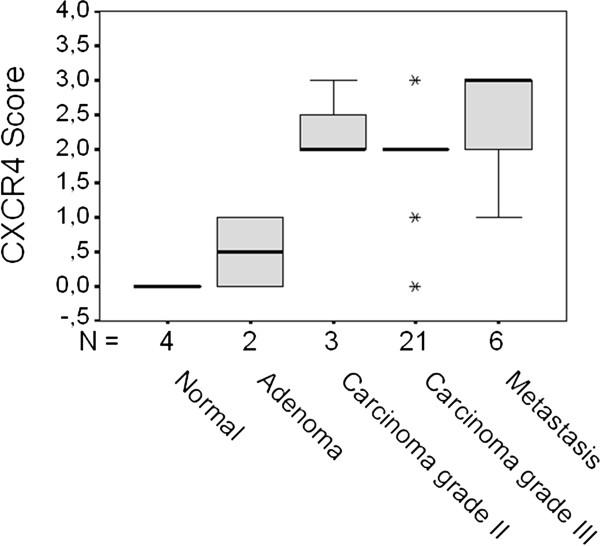
**CXCR4 score in feline mammary carcinomas**. Box plots represent the distribution of CXCR4 score in different feline mammary carcinoma histological types. A statistically significant relationship (Pearson's Chi sqr. test) was observed between the increase of CXCR4 scores from normal to benign, malignant and metastatic mammary tissues. Each box shows the median, quartiles, and extreme values within a group.

Figure 3 summarized the distribution of CXCR4 scores reported in Table 1, among all different grades of tumours, metastasis and four normal tissues. Box plots shows the median CXCR4 score for each group: 0 for normal mammary glands and 0.5+ for benign lesions, median values increased in grade II and grade III malignant tissues (median sore 2+) while metastases expressed the highest score. (median value = 3+). By analyzing the frequency distribution of CXCR4 expression score in the different groups we observed a statistically significant association between these two variables (Pearson Chi-square = 34.96, p = 0.0005).

Interestingly, 4 out of 6 metastatic tumours appeared to have a stronger CXCR4 expression as compared to the respective primary tumours (increase of the CXCR4 score, from 2+ to 3+ in cat n. 4, 5,12 and 23, Table 1). Nevertheless, in two metastatic sites, the neoplastic cells showed either a lighter immunoreaction if compared with the respective primary tumour (cat n. 8) or no changes (cat n.2).

### SDF-1/CXCR4 role in primary cultures of feline mammary carcinoma

**Figure 4:**
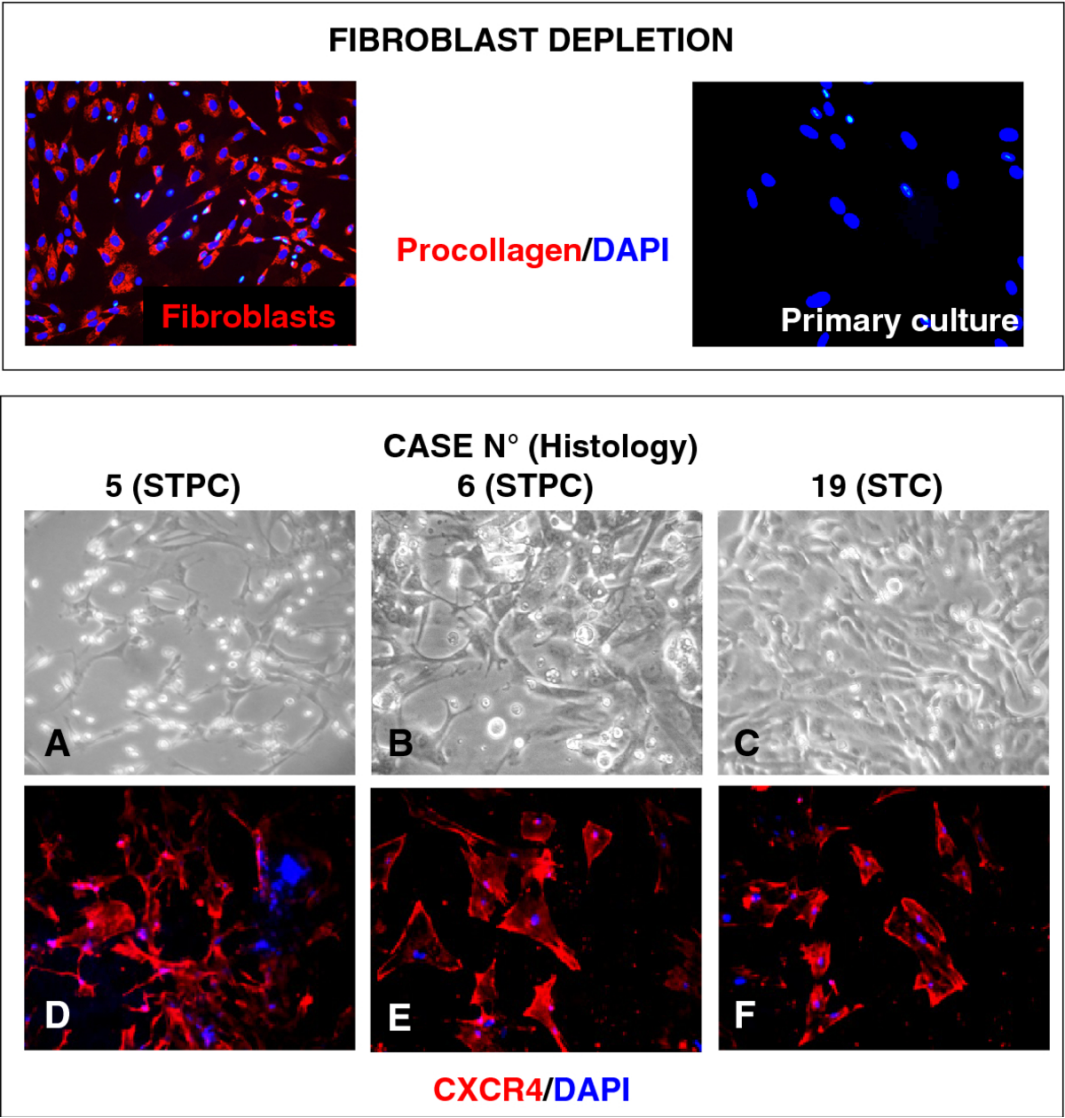
**CXCR4 expression in primary cultures of feline mammary carcinoma**. **Upper panels**. Representative immunofluorescence staining using anti-procollagen I (red) of fibroblast and purified feline mammary carcinoma primary cultures, after immunomagnetic fibroblast separation. DAPI-counterstained nuclei in blue. No fibroblast contamination is observed in the tumor cell cultures. **Lower panel. A-B-C**. Phase-contrast microscopy observation of the cultured cells derived from feline mammary gland tumours with an epithelioid morphology with some elongated spindle-shaped cells growing in monolayer (original magnification 10×) **Panels D-E-F: **immunofluorescence detection of CXCR4 expression (red) in primary cultures (DAPI-counterstained nuclei in blue). Original magnification 20×.

To investigate the biological role of CXCR4 expression in feline mammary carcinoma, fibroblast-free, primary cell cultures were obtained from 6 primary carcinomas (cat n. 5, 6, 19, 22, 23 and 24). *In vitro *feline mammary cancer cells appeared as adherent monolayers with spindle-like shape (Figure 4, panels A, B, C). The expression of CXCR4 in each culture was verified by IF staining, confirming the immunopositivity of mammary carcinoma primary cells (Figure 4, panels D, E, F), as in the tissue of origin.

The absence of contaminant fibroblasts, which can interfere with both immunofluorescence and proliferation studies, was demonstrated by the lack of pro-collagen expression in the mammary carcinoma cell cultures, while isolated tumour fibroblasts, used as positive controls, showed a strong staining (Figure 4, upper panels).

**Figure 5:**
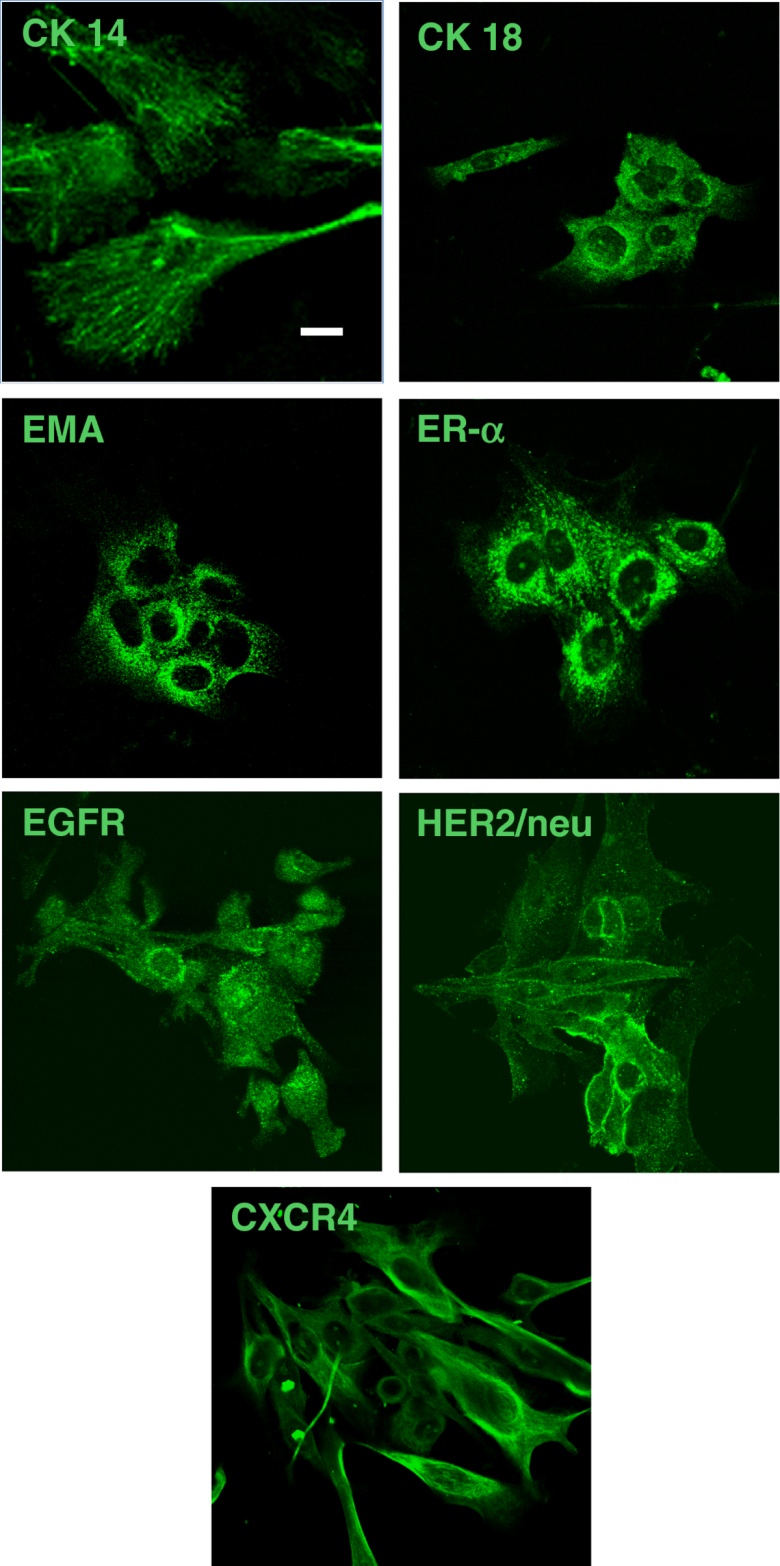
**Immunofluorescence analysis of primary cultures for mammary malignant epithelial cell markers**. Cells were stained with relevant markers (CK14, CK18, EMA, ER-α) for epithelial mammary cells frequently over-expressed proteins in malignant mammary tumors (HER2/neu, EGFR): immunopositivity demonstrates the presence in feline primary carcinoma cultures the presence of malignant epithelial mammary cells. Representative confocal microscopy images are reported. Bar 25 μM, original magnification 60×.

Beside CXCR4 expression, a phenotypic characterization of the cells from the primary cultures was performed, by analyzing the expression of common mammary lineage markers (CK14, CK18, EMA, ER-α), or proteins (EGFR and HER-2/neu) overexpressed in mammary carcinoma conferring malignant behaviour (Figure 5). As expected, feline mammary carcinoma cells from all tumour tested, homogeneously expressed the mammary epithelial marker EMA. Moreover, *in vitro *cultures abundantly expressed CK14 (100% of cultures) and, in variable proportion of cells, CK18 (83% of cultures). In addition, we include the evaluation of ER-α immunocytofluorescence to define its pattern of expression in feline mammary cancer cells, detecting different levels of positivity, but generally low or weak (60% of immunopositivity). As far as HER2/neu and EGFR expression in feline primary cultures, both proteins were expressed in all mammary carcinoma cultures. Representative results from immunocytofluorescence are reported in Figure 5, including the CXCR4 positivity.

**Figure 6:**
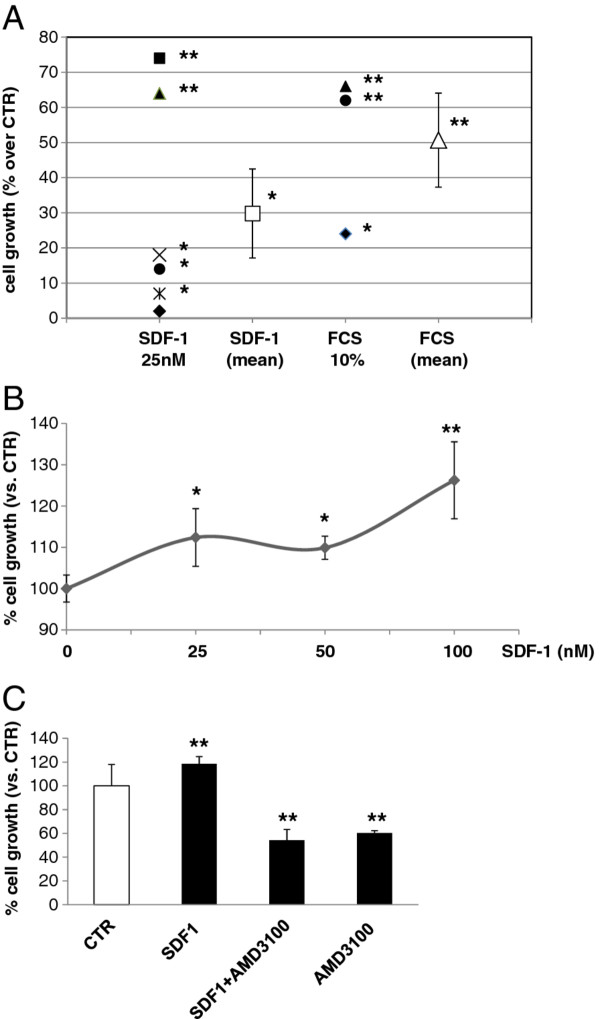
**SDF-1 growth stimulation on feline mammary carcinoma primary cultures**. **A**. Scatterplot represents the individual and mean values of increase of growth rate in 6 primary cultures stimulated with SDF-1 (25 nM) or 10% FCS for 24 h, evaluated by MTT assay. *P < 0.05, **P < 0.01 (*vs*. serum-starved control). **B**. Dose-response curve of SDF-1 treatment for 24 h. *P < 0.05, **P < 0.01 (vs. untreated serum- starved control) evaluated by MTT assay. **C**. Effects of CXC4 inhibition by the antagonist AMD3100 on SDF-1 cell growth stimulation evaluated by MTT assay. Cells were treated with SDF-1 (25 nM) for 24 h in the absence or presence of AMD3100 (10 μM). AMD3100 significantly reverts SDF-1 growth stimulus and affects basal proliferation. **P < 0.01 (vs. untreated serum- starved control).

In order to analyze the proliferative effects induced by CXCR4 activation, the obtained mammary carcinoma primary cultures were starved for 24 h and treated with 25 nM SDF-1 for further 24 h (the concentration was chosen as the maximal growth stimulatory effect obtained in previous experiments on human breast cancer cell lines [19]), and then MTT cell viability assays were performed. SDF-1 exposure induced a statistically significant proliferative stimulus in 5 out of 6 feline mammary cultures as compared to serum-starved untreated controls, with a mean increase of cell growth of +30%, p < 0.05 (Figure 6A). To discriminate the level of growth-arrest induced by serum deprivation, in a subgroup of 3 cultures the SDF-1 stimulus was compared to complete medium (10% FCS) effects: serum was able to significantly recover the proliferative rate of cells of about +50% over the corresponding starved cultures. Thus, SDF-1 significantly promotes feline mammary carcinoma cell growth, although with individual differences among cultures from different tumors, that is in the same order of magnitude, although lower, than that induced by FCS. To characterize the mitogenic effects of SDF-1, a dose-response curve (25, 50 and 100 nM) was performed in one primary culture (cat n. 22). The chemokine showed a dose-dependent effect with all the concentrations tested able to induce a significant growth stimulation, that was maximal at 100 nM.

To demonstrate that SDF-1 effects occurred through CXCR4 expressed in feline mammary carcinoma cells, we exposed cells to the CXCR4 antagonist AMD3100. AMD3100 pre-treatment, was able to revert the SDF-1 increase in cell proliferation, confirming that CXCR4 is mediating SDF-1 effects in these cells. In addition, we observed that AMD3100 significantly inhibits basal cell growth, possibly implying the occurrence of a constitutive role of CXCR4 in cell proliferation.

## Discussion

Several studies demonstrated that: a) the expression of chemokine receptors in neoplastic cells is not random; b) CXCR4 is the most widely expressed chemokine receptor in most tumours; c) the effects of SDF-1 on CXCR4-expressing cancer cells are pleiotropic [31]. In particular, SDF-1/CXCR4 axis is enrolled in many functional aspects of tumour progression, such as angiogenesis, site-specific metastasization, proliferation and survival of neoplastic cells [2,32]. It is also recognized that metastastic behaviour of cancer cells may be reinforced by hypoxia, a condition that induce up-regulation of CXCR4 expression mediated by hypoxia-inducible factor-1a (HIF-1α) [33,34]. SDF-1/CXCR4 axis is involved in the promotion of angiogenesis, local cell proliferation and migration of cancer cells to the metastatic sites in many different kinds of cancers as those of breast [21,35], lung [36], ovarian [1,37], renal [38], prostate [39], and neuroblastoma [40]. Moreover, it seems relevant to remind that CXCR4 expression has been associated to a worse prognosis in human tumours, and then, in the future, it could be also used as prognostic indicator [41].

In contrast with the large number of studies in human oncology, there are only few scientific articles supporting the role of SDF-1/CXCR4 axis in spontaneous tumours of domestic animals. Tanabe and colleagues [26] observed that the receptor's mRNA is not identifiable in mammary tissue of healthy cats. However, they found CXCR4 mRNA in area adjacent to necrotic tissue, surrounding blood vessels and in cells infiltrating the lymphatic tissue in 72.3% of 65 samples. In the same study, the authors observed a statistically significant relationship between infiltration of neoplastic cells in lymphatic and CXCR4 expression [26], although no relationship was observed between CXCR4 expression and one-year survival time of the cats included in the study [26]. In another study, higher expression of CXCR4 mRNA in metastatic cells as compared with cells from primary tumours was reported, altogether with the observation that neoplastic cells from feline mammary carcinomas express more CXCR4 than non-neoplastic mammary tissues [25].

In the present study we demonstrated, for the first time, a high CXCR4 expression in several feline high grade mammary carcinoma, evaluated at protein level by immunohistochemistry. Interestingly, although the limited number of benign and metastatic lesions, but including normal tissues, we observed a statistically significant correlation between CXCR4 score levels and the increase of tumor grade or metastases. However, without drawing a definitive conclusion due to the need of higher numbers, the present results showing absence or low CXCR4 expression in normal and benign mammary tissues may suggest a specificity of CXCR4 expression for mammary carcinomas and metastases.

As far as the malignant primary tumours CXCR expression, although we observed some variability in the intensity of the anti-CXCR4 immunoreaction, only occasionally we detected variation in the percentage of positive cells. Moreover, although a large number of samples will be required to perform an appropriate statistical analysis, in this study the majority of metastastic lesions (5/6) displayed a higher expression compared to cells within the respective primary lesion. These observations, at least in part, support the results reported in the literature for breast cancer in women and in feline mammary carcinoma [42].

The interest in researching on SDF-1/CXCR4 pathways is also oriented to discover 'new chemical entities' able to block this mechanism and specifically inhibit CXCR4 functions. Actually, there are some examples published in the international literature reporting the possible use of CXCR4 inhibitors. The latter have been used to reduce growth and metastasis of head and neck cancers and intracranial growth of brain tumours [43,44]. These results have been also supported by *in vitro *experiments that proved the efficacy of CXCR4 inhibitors in blocking SDF-1/CXCR4-mediated proliferation and migration in breast cancer and lymphoblastic leukaemia [19,45]. In veterinary oncology, Oonuma and collaborators [25] found a similar response *in vitro *using established feline mammary carcinoma cell lines incubated with CXCR4 antagonists.

Here, besides the expression of CXCR4 in feline mammary tumours, we also investigated the functional role of this receptor in mediating proliferative signals. *In vitro*, 5 out of 6 analyzed primary cultures of mammary carcinoma cells showed significant increased cell proliferation in response to CXCR4 activation by nanomolar concentrations of SDF-1. The effect was quantitatively variable but reached, on the average a values similar to that induced by FCS, thus confirming that SDF-1/CXCR4 axis is an important signalling pathway involved in feline mammary cell proliferation. Interestingly, the use of the CXCR4 antagonist AMD3100, while confirming the specificity of SDF-1 effects *in vitro *through CXCR4, showed a reduction also in basal proliferation rate. Thus, it could be hypothesized that a basal SDF-1 secretion leads to autocrine CXCR4 activation in some tumor cell culture. This observation may also explain why in few cultures a lower proliferation is induced by exogenous SDF-1 that could be masked by the autocrine CXCR4 activation. A similar different response, related to constitutive autocrine SDF-1 effect, was recently demonstrated in human pituitary adenoma cell cultures *in vitro *[46,47]. A larger number of tumors have to be analysed to confirm this hypothesis.

These data, although preliminary, strongly suggest that CXCR4 activity controls mammary carcinoma cell proliferation in cats as it does in humans, and propose that pharmacological inhibition of this receptor may represent an innovative approach for this kind of tumours. Moreover, our data propose that primary feline can reflect and maintain *in vitro *the phenotype of epithelial malignant carcinoma mammary cells and are potential suitable experimental models for assessing the biological activity of novel molecules with antitumour effects.

In the past, several studies reported the relevance of naturally occurring cancers in domestic animals as model for study human cancer biology and translational therapeutics [48,49]. Among other tumours, mammary carcinomas show a relatively high similarity with the human counterpart as far biological behaviour, clinical course and responses to cytotoxic agents. The development of *in vitro *models of feline mammary carcinomas may represent a relevant tool to identify novel molecular pharmacological targets to be used in veterinary setting and possibly extended to humans.

## Conclusions

With the increase in the development of novel therapeutic agents feline mammary carcinomas can provide a useful model to test new drugs, as far as efficacy and toxicity, including novel CXCR4 antagonists.

In conclusion, our results suggest that routine evaluation of CXCR4 in feline mammary neoplastic lesions might be useful for selection of cases which may be treated with targeted chemotherapy. However, further investigations are required to better evaluate the potential role of CXCR4 as prognostic factor and as a target for novel chemotherapeutic agents.

## Methods

### Tumor samples and tissue processing

Twenty-six animals were included in this study. Feline mammary lesions (n = 33) and metastasis (n = 6) collected at the time of surgical excision, were obtained from the histopathology service of the National Reference Centre for Veterinary and Comparative Oncology of the "Istituto Zooprofilattico Sperimentale del Piemonte, Liguria e Valle D'Aosta" of Genova, Italy. Four normal mammary tissue samples were obtained from unaffected glands resected during tumor surgery.

All the collected tissues were fixed in 10% buffered formalin and embedded in paraffin. Tissue sections (3 μm) were stained by standard haematoxylin and eosin methods for histological examination and classified according to the World Health Organization (WHO) Histological Classification of Mammary Tumors of the Dog and Cat [50]. Histological grade was defined accordingly to the histological grading system of canine and feline mammary carcinoma [51]. Briefly, histological grade was defined taking account of tubule formation (from 1 point if the tissue section has well-marked tubule formation to 3 points if there are very few or no tubules), hyperchromatism and mitoses (1 point if only an occasional hyperchromatic or mitotic figures per high-power field are seen; 2 points if there are two or three such figures, and 3 points if the number is higher), and irregular size and shape of nuclei (from 1 point if nuclei are fairly uniform in size, shape and staining and 3 points if pleiomorphism is marked). Finally, the score of all three components (tubule formation, mitotic count, and nuclear pleiomorphism), were added together to give the grade. The combined histological grade with final scores of 3-5 were designed grade I or low grade tumours, scores of 6 or 7 were classified grade II or intermediate grade tumours, and 8 or 9 were classified as grade III or high grade tumours.

### Immunohistochemistry

Immunohistochemical analysis was performed using a rabbit anti-CXCR4 antibody (C3116, Sigma-Aldrich, Milano. Italy). Tissue sections (3 μm) were deparaffinized in xylene, rehydrated in a graded series of ethanol solutions, placed in Coplin jars containing a citrate solution 1:10 in distilled water (Target Retrieval Solution, citrate pH 6.0, Dako Cytomation, Glostrup, Denmark) and heated in a water bath at 98°C for 20 min to unmask the antigen. After the eating step, slides were allowed to cool for 15 min under running water and washed with a solution 1:10 of Tris-buffered saline (TBS) in distilled water (TBS solution, 10×, pH 7.4, Bioptica, Milano, Italy). Endogenous peroxidase activity was quenched by immersion in solution of 3% hydrogen peroxide in distilled water for 30 min, followed by several rinses in TBS. Non-specific binding was blocked by incubation with 5% bovine serum albumin (BSA, Albumin Fraction V, pH 7.0, Applichem, Darmstadt, Germany) in TBS for 30 minutes at r.t. Slides were incubated overnight with anti-CXCR4 diluted 1:500 in Antibody Diluent with Background reducing Components (Dako North America Inc., Carpinteria, CA, USA). The slides were rinsed in TBS (two times for 4 min) and then incubated with secondary antibody REAL Envision Detection System Peroxidase/DAB+, mouse/rabbit (Dako) according to the manufacturer's instruction. The slides were rinsed in TBS (two times for 4 min) and then stained with 3-3'-diaminobenzide tetrahydrochloride accordingly with manufacture's instruction. Sections were counterstained with Mayer's haematoxylin solution. Negative controls were run in parallel, substituting the primary antibody with 5% BSA in TBS or rabbit IgG (Sigma-Aldrich, see Supplementary Figure S1, panel A and B, respectively). To verify the specificity and cross-reactivity of the rabbit-anti CXCR4 antibody for feline mammary tissues, in some cases we run parallel immunohistochemical staining with the Monoclonal Mouse IgG2B CXCR4 antibody from R&D Systems, (Minneapolis, MN, USA) that we also used in immunofluorescence experiments and cross-reacts with both human and feline CXCR4 (Supplementary Figure S1, C panel D show the comparison between the 2 antibodies). As positive control, 10% neutral-buffered formalin fixed tissue sections of human breast carcinoma were used (data not shown). Tissue sections were evaluated by light microscopy (Nikon Coolscope, Nikon, Firenze, Italy) to determine CXCR4 immunoreaction and the cellular localization of positive immunolabelling. Both intensity of the resulted immunoreaction and percentage of positive cells were evaluated taking into account previous experiences published by two different authors [52,53]. Tumour cells with brown cytoplasm were considered positive and staining intensity was classified as: weak brown (1+); moderate brown (2+) and strong brown (3+). Percentage of stained tumour cells, obtained evaluating at least 1000 neoplastic cells in 10 high-power fields for each tissue section, was categorized into four classes: 0 = negative, 1 = < 10%; 2 = 10-50%; 3 = > 50%. Multiplication of intensity and percentage scores (staining index) was utilized to determine the following results: 0, 1, 2, 3, 4, 6, and 9. The final results were then categorized as 0, 1+, 2+ and 3+ as reported below:

a. Staining index 0: (0 = 0) and 1(1 × 1 = 1) were considered negative (0)

b. Staining index 2: (2 × 1 = 2) and 3 (3 × 1 = 3) were considered (1+)

c. Staining index 4: (2 × 2 = 4) and 6 (2 × 3 = 6) were considered (2+)

d. Staining index 9: (3 × 3 = 9) was considered (3+)

### Primary cell cultures

Tumor cells were isolated from 6 primary feline mammary carcinomas (Table 1, cat n. 5, 6 and, 19, 22, 23 and 24) immediately after surgical removal. A portion of fresh tumour tissue was excised and saved in sterile tubes with D-MEM medium (Lonza, Verviers, Belgium) supplemented with penicillin/streptomycin (200 U/ml; Lonza), amphotericin B (250 ng/ml, Sigma-Aldrich) to avoid bacterial and fungi contamination. Samples were washed PBS to eliminate blood and debris eventually present, dissected to take off skin or other non-tumour tissues and mechanically disaggregated. Cell suspension was filtered through a 70 μm cell mesh (BD Biosciences, Bedford, MA, USA) recovering single cells by centrifugation. The isolated cells were seeded into T25 flasks in D-MEM culture medium containing 10% FCS, 100 U/ml penicillin/streptomycin and 2 mM glutamine (Lonza). To avoid fibroblast contamination and overgrowth in culture, FMC cells were purified by immunomagnetic separation with anti-fibroblast microbeads (MACS, Miltenyi Biotec, Bologna, Italy) as reported [54]. Pro-collagen (anti-procollagen type I, SP1.D8, Developmental Studies Hybridoma Bank, Iowa City, IA, USA) immunofluorescent staining was performed to verify the absence of fibroblast contamination and overgrowth in culture. Exemplificative images of the absence of fibroblast contamination in mammary tumour cells are reported in Figure 4 (upper panel). Cells were maintained at 37°C in a humidified incubator at 5% CO_2_. Individual culture flasks were observed daily and photographed by a phase-contrast microscope for the presence of cell monolayers and morphologies.

### Immunocytofluorescence

Immunofluorescence (IF) detection was performed as reported [46]. Briefly, feline mammary carcinoma cells were grown on coverslips, fixed with 4% paraformaldehyde in PBS and stained with CXCR4 (R&D Systems, Minneapolis, MN, USA), epithelial membrane antigen (EMA), cytokeratins 14 (CK14) and 18 (CK18), ER-α, Her2/neu (Dako), EGFR (Cell Signaling, Beverly, MA, USA). Cells were labelled with fluorochrome-conjugated secondary antibody (red-fluorescent Alexa Fluor 568 or green-fluorescent Alexa Fluor 488 dyes, Molecular Probes Invitrogen, Milano, Italy) for 1 h, nuclei were counterstained with 4',6-diamidino-2-phenylindole (DAPI, Sigma-Aldrich) and mounted with Mowiol. Negative controls were included in the experiments by omitting the primary antibodies. IF slides were visualized and photographed with a DM2500 microscope (Leica Microsystems, Wetzlar, Germany) equipped with a DFC350FX digital camera (Leica Microsystems) or confocal laser scanning microscopy (Bio-Rad MRC 1024 ES).

### MTT assay

Cell viability was determined by the reduction of 3-(4,5-dimethylthiazol-2-yl)-2,5-diphenyltetrazolium bromide (MTT, Sigma-Aldrich) [55]. After seven days in vitro to expand the culture, cells were seeded in 24-well plates in complete medium, serum-starved for 24 h and then treated with 25 nM SDF-1 (Immunological Sciences, Roma, Italy) or 10% FCS for further 24 h. The AMD3100 (Sigma-Aldrich) CXCR4 antagonist was added to cultures at 10 μM for 24 h in the presence or absence of SDF-1. MTT solution (0.25 mg/ml in PBS, final concentration) was added for 4 h at 37°C. optical density (O.D.) was measured spectrophotometrically at 570 nm. Data are expressed as mean ± S.D. and referred to the 100% value of serum-starved control samples.

### Statistical analysis

Statistical analysis was performed with SPSS 9.0 software (SPSS Inc., Chicago, USA) using ANOVA, cross-tabulation and Pearson chi-square test, when appropriate.

P ≤ 0.05 was accepted as statistically significant.

## Authors' contributions

AF: conceived the study and drafted the manuscript; CP: participated in the design of the study, carried out the histopathological diagnoses and drafted the manuscript; AR: coordinated the collection of fresh tissue samples and clinical information, carried out the histopathological diagnoses and supervised the immunohistochemical staining and scoring process; CC, GV: carried out immunohistochemistry; RW and ST: performed primary culture isolation and proliferation assays; FB: performed immunofluorescence and statistical analysis and helped to draft the manuscript; TF: conceived and supervised the experimental plan and drafted the manuscript. All authors read and approved the final manuscript.

## Supplementary Material

Additional file 1 **Figure S1 Immunohistochemical controls for CXCR4 expression in feline mammary carcinomas**. **A**. Negative control obtained by omitting the rabbit primary antibody against CXCR4. **B**. Negative control obtained by substituting the primary antibody with rabbit IgG. **C**. CXCR4 staining with rabbit anti-CXCR4 antibody (Sigma-Aldrich). **D**. CXCR4 staining with rabbit anti-CXCR4 antibody (R&D Systems). Tissue images derived from a tubulopapillary carcinoma, bar = 25 microm, original magnification 40×. Click here for file
