# Supplementary material for: CXCR4 expression in feline mammary carcinoma cells: evidence of a proliferative role for the SDF-1/CXCR4 axis
Source: BMC Vet Res. 2012 Mar 14;8:27. doi: 10.1186/1746-6148-8-27 (PMC3364888; doi:10.1186/1746-6148-8-27)
Supplement: Additional file 1 — Figure S1 Immunohistochemical controls for CXCR4 expression in feline mammary carcinomas. A. Negative control obtained by omitting the rabbit primary antibody against CXCR4. B. Negative control obtained by substituting the primary antibody with rabbit IgG. C. CXCR4 staining with rabbit anti-CXCR4 antibody (Sigma-Aldrich). D. CXCR4 staining with rabbit anti-CXCR4 antibody (R&D Systems). Tissue images derived from a tubulopapillary carcinoma, bar = 25 microm, original magnification 40×. [file 1746-6148-8-27-S1.PPT]

## Slide 1
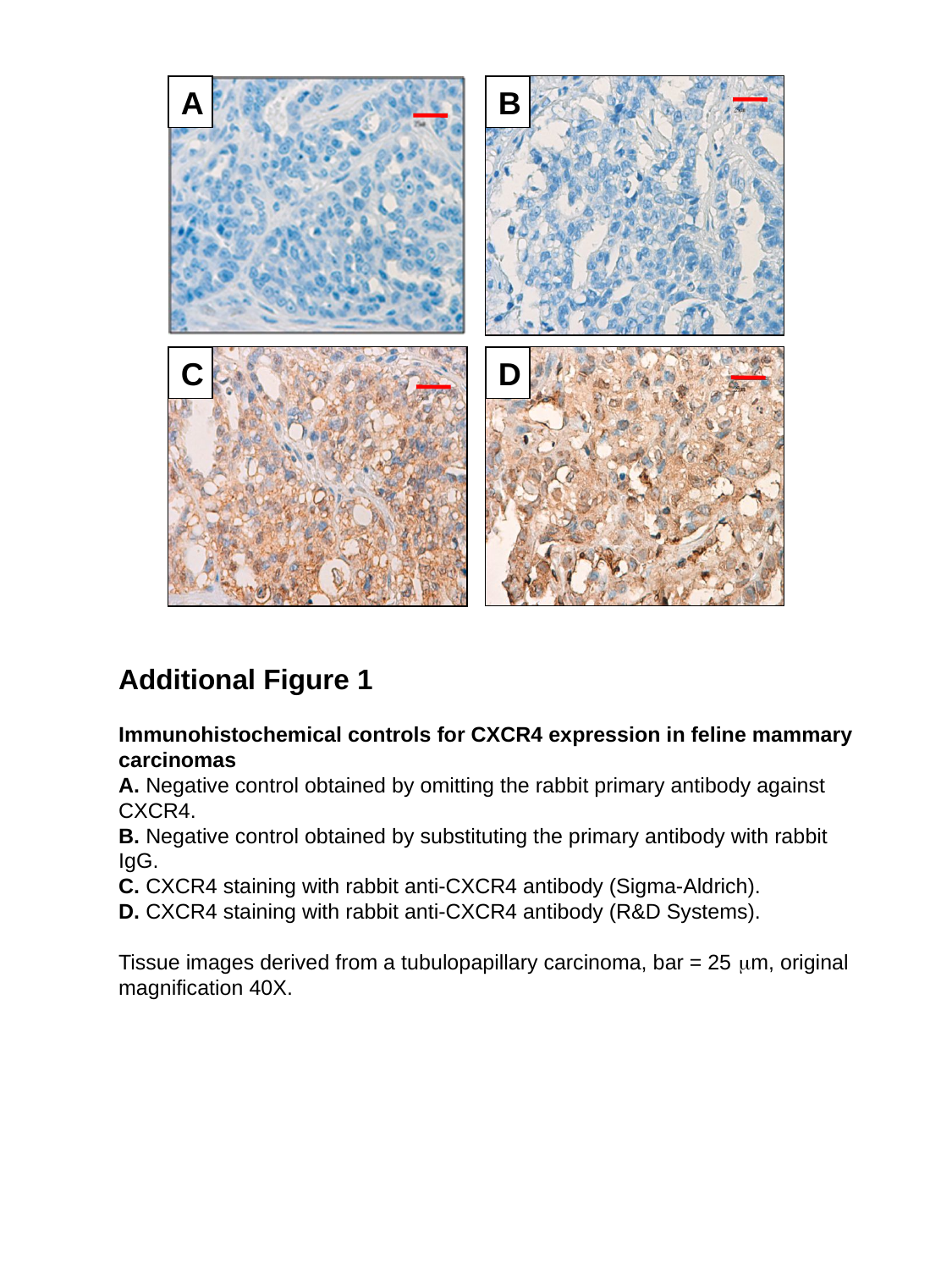

A
B
C
D
Additional Figure 1
Immunohistochemical controls for CXCR4 expression in feline mammary carcinomas
A. Negative control obtained by omitting the rabbit primary antibody against CXCR4.
B. Negative control obtained by substituting the primary antibody with rabbit IgG.
C. CXCR4 staining with rabbit anti-CXCR4 antibody (Sigma-Aldrich).
D. CXCR4 staining with rabbit anti-CXCR4 antibody (R&D Systems).
Tissue images derived from a tubulopapillary carcinoma, bar = 25 μm, original magnification 40X.
